# Is a Central Sediment Sample Sufficient? Exploring Spatial and Temporal Microbial Diversity in a Small Lake

**DOI:** 10.3390/toxins12090580

**Published:** 2020-09-09

**Authors:** Barbara Weisbrod, Susanna A. Wood, Konstanze Steiner, Ruby Whyte-Wilding, Jonathan Puddick, Olivier Laroche, Daniel R. Dietrich

**Affiliations:** 1 Human and Environmental Toxicology, Department of Biology, University of Konstanz, Universitätsstrasse 10, 78457 Konstanz, Germany; 2 Cawthron Institute, 98 Halifax Street East, Nelson 7010, New Zealand; Susie.Wood@cawthron.org.nz (S.A.W.); Konstanze.Steiner@cawthron.org.nz (K.S.); rubyw.wilding@gmail.com (R.W.-W.); Jonathan.Puddick@cawthron.org.nz (J.P.); Olivier.Laroche@cawthron.org.nz (O.L.)

**Keywords:** cyanobacteria, earthquakes, harmful algal blooms, microcystin, sediment, sediment cores

## Abstract

(1) Background: Paleolimnological studies use sediment cores to explore long-term changes in lake ecology, including occurrences of harmful cyanobacterial blooms. Most studies are based on single cores, assuming this is representative of the whole lake, but data on small-scale spatial variability of microbial communities in lake sediment are scarce. (2) Methods: Surface sediments (top 0.5 cm) from 12 sites (*n* = 36) and two sediment cores were collected in Lake Rotorua (New Zealand). Bacterial community (16S rRNA metabarcoding), *Microcystis* specific 16S rRNA, microcystin synthetase gene E (*mcyE*) and microcystins (MCs) were assessed. Radionuclide measurements (^210^Pb, ^137^Cs) were used to date sediments. (3) Results: Bacterial community, based on relative abundances, differed significantly between surface sediment sites (*p* < 0.001) but the majority of bacterial amplicon sequence variants (88.8%) were shared. Despite intense MC producing *Microcystis* blooms in the past, no *Microcystis* specific 16S rRNA, *mcyE* and MCs were found in surface sediments but occurred deeper in sediment cores (approximately 1950′s). ^210^Pb measurements showed a disturbed profile, similar to patterns previously observed, as a result of earthquakes. (4) Conclusions: A single sediment core can capture dominant microbial communities. Toxin producing *Microcystis* blooms are a recent phenomenon in Lake Rotorua. We posit that the absence of *Microcystis* from the surface sediments is a consequence of the Kaikoura earthquake two years prior to our sampling.

## 1. Introduction

Lake ecosystems worldwide are experiencing dramatic changes in water quality, nutrient concentrations and phytoplankton community composition due to global warming, eutrophication and introductions of non-native species [1,2]. Consequently, the occurrence of harmful cyanobacterial blooms has increased significantly [3,4]. Many bloom-forming species produce cyanotoxins, which have neurotoxic, cytotoxic, and hepatotoxic effects on organisms [5]. Contact with or ingestion of water contaminated with cyanotoxins can lead to poisoning, or death of humans, pets, stock, and wildlife [5]. Amongst the cyanobacterial toxins, the cyclic heptapeptide microcystins (MCs) are the most frequently found [6,7,8,9,10].

MCs are potent inhibitors of eukaryotic protein phosphatases resulting in a wide range of organ toxicities, but primarily causing liver damage [11]. Cyanobacteria genera known to produce MCs include *Planktothrix*, *Oscillatoria*, *Dolichospermum* and most prominently *Microcystis* [5]. At least 271 structural variants of MCs have been described [12]. MCs are synthesized non-ribosomally by a large multifunctional enzyme complex which contains both non-ribosomal peptide synthetase and polyketide synthase domains [13]. The *mcy* gene cluster (*mcyA-J*) encoding these biosynthetic enzymes has been employed as the target region for the molecular detection of potentially toxin producing cyanobacteria strains in water and sediment [6,14,15].

Considerable effort has been invested in developing management and restoration plans for water bodies experiencing cyanobacterial blooms [16,17,18]. Beyond the need for effective bloom mitigation strategies, there is an increasing demand for understanding how lake ecosystems and their catchments have changed historically and how this might correlate with increasing prevalence of cyanobacterial blooms. Unfortunately, long-term monitoring data are scarce. An emerging technique that can contribute historic data is the application of molecular biological methods to sediment core analysis [19]. Employing molecular techniques, e.g., metabarcoding and droplet digital PCR (ddPCR), allows reconstruction of historical cyanobacteria communities using phylogenetic marker genes like the 16S ribosomal RNA (16S rRNA) but also functional genes (such as the *mcy* gene cluster) identified in sediment samples [6,7]. Accumulation of cyanobacterial DNA and toxins in sediments occurs via natural cell sedimentation during and after blooms [20]. This is especially true for *Microcystis* sp. that overwinter in surface sediments [21,22].

Both cyanobacterial DNA and MCs are thought to be well preserved in sediments, and both have been detected in sediment layers dating back over 200 years [6,7,8,9,10]. Most paleolimnological studies on cyanobacteria [6] but also eukaryotes [23,24], collect sediment cores from a single central site or deepest point in a lake, assuming a relatively even distribution across the lake. However, during a bloom cell densities of buoyant cyanobacteria, e.g., *Microcystis* spp., are highly dependent on wind and current drift, hence there is often high spatial variability across a lake surface [25,26,27]. Cell and toxin concentrations at one site can change dramatically within short time periods. Given the spatial patchiness of cyanobacterial blooms, and the accumulation of buoyant scums along shorelines, it is likely that cyanobacterial cells and toxins may also be unevenly distributed on lake sediment. In addition, MC sediment concentration resulting from surface blooms are likely influenced by a complex interplay of microbial degradation, thermal decomposition and photolysis via UV radiation which can all vary within different sites of the same lake [28,29]. Therefore, there is an uncertainty as to whether single core samples are representative of the whole lake and provide an accurate record of bloom history. Most research regarding spatial distribution of sediment microbial communities has been undertaken on intermediate (different lakes) [30] to global (thousands of kilometers) scales [31,32,33]. Hence, a better understanding of small-scale (tens to hundreds of meters) distribution patterns within a lake is required to ensure a robust interpretation of paleolimnological results.

This study focuses on Lake Rotorua, a small hypertrophic lake in the northeast of the South Island of New Zealand [34]. The lake has experienced cyanobacterial blooms since at least the 1970s with *Dolichospermum*, *Aphanizomenon* and MC-producing *Microcystis* present in high abundances. However, there is uncertainty as to whether these species have always been present in the lake and when blooms begun. The aims of this study were to: (i) determine the spatial variability in bacterial and cyanobacterial species in the surface sediment of Lake Rotorua, with a focus on the abundance of *Microcystis*, MC synthetase gene E (*mcyE*) copy numbers and MCs; and (ii) explore the historical bacterial (in particular cyanobacteria) composition in two sediment cores.

## 2. Results

### 2.1. Physicochemical Parameters

Temperature, pH and salinity did not differ markedly between sampling sites (Table S1). Dissolved oxygen concentration varied between 0.4 and 7.6 mg L^−1^ and was highest in the embayment (S01, 7.6 and S02, 6.3 mg L^−1^) and at one littoral site (S08, 6.6 mg L^−1^; Table S1).

### 2.2. Surface Sediments

#### 2.2.1. Detection of *Microcystis* Sp. Specific 16S rRNA and *mcyE*

**Figure 1:**
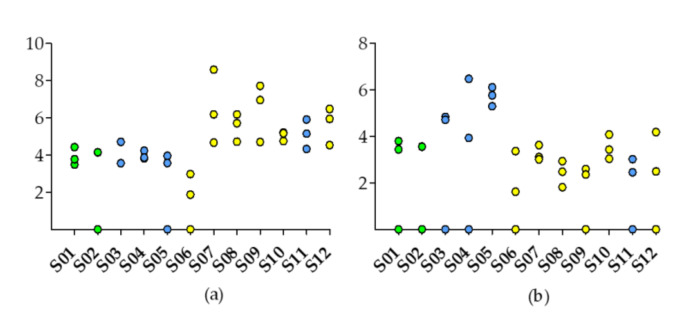
Abundance of *Microcystis* specific, (**a**) 16S rRNA gene, and (**b**) *mcyE* gene in surface sediment samples (S01–S12). Sample sites are indicated by color (embayment = green, mid-lake = blue, littoral = yellow). Data are log(x + 1)-transformed.

All samples, besides the littoral sample S06, contained low levels of the *Microcystis* sp. 16S rRNA gene (Figure 1a). The littoral samples S07, S09 and S12 contained more than 10^6^ gene copy numbers g^−1^ sediment (Figure 1a). The *mcyE* gene copy numbers were relatively high in the three mid-lake samples (S03, S04, S05) (69,443–1.3 × 10^6^ gene copy numbers g^−1^ sediment), low (≤15,565 gene copy numbers g^−1^ sediment) in samples S01–02, S06–07, S10 and S12, and no copies were detected in samples S08, S09 and S11 (Figure 1b). As a single copy gene, *mcyE* gene copy numbers should be present at a lower concentration compared to the *Microcystis* 16S rRNA copy numbers. This was observed for samples S01-03, and S06-12, while higher *mcyE* gene copy numbers than corresponding 16S rRNA copy numbers were observed for samples S04 and S05.

#### 2.2.2. Bacterial Community

**Figure 2:**
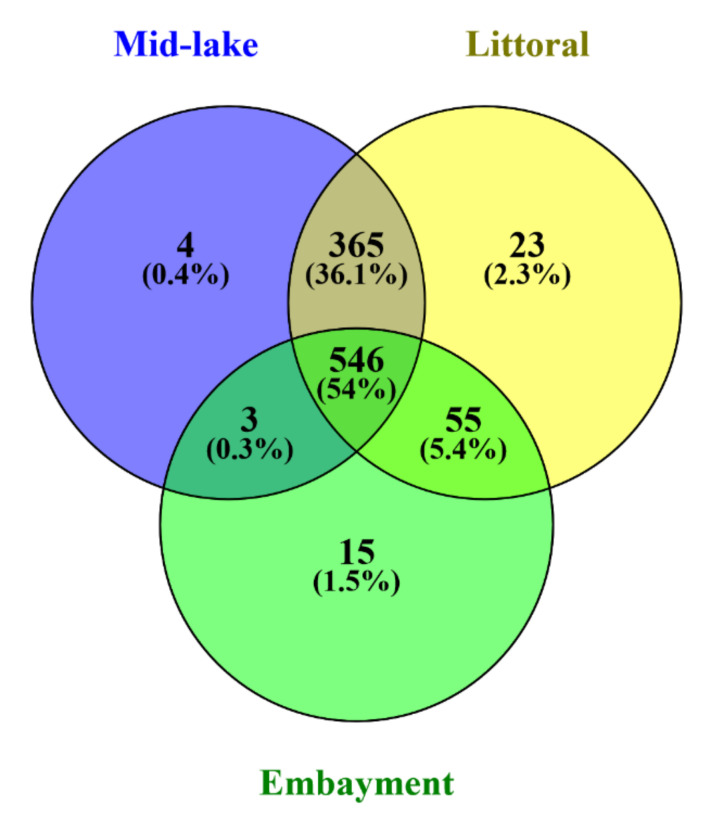
Venn diagram illustrating the unique and shared amplicon sequence variants (ASVs) between the three sampling sites of surface sediments.

**Figure 3:**
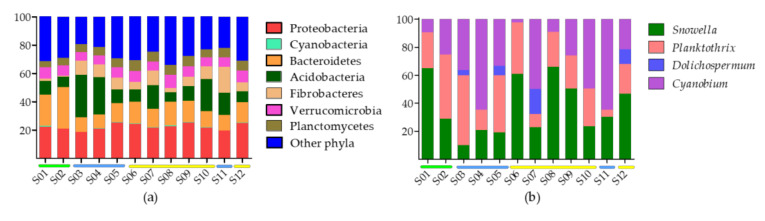
Bacterial community of surface sediment samples (S01–S12) based on 16S rRNA sequences. Relative abundance of; (**a**) cyanobacteria and the seven most abundant bacteria phyla the in the total bacterial community, and (**b**) cyanobacteria genera in the cyanobacteria portion of the data. Sample sites are indicated by colour (embayment = green, mid-lake = blue, littoral = yellow).

The total number of unique 16S rRNA amplicon sequence variants (ASVs) after quality-filtering and pre-processing was 1011. Negative controls contained 372 reads assigned to 12 ASVs. These were excluded when ASV that accounted for >0.01% of reads were removed. A total of 546 ASVs (54%) were shared between embayment, littoral and mid-lake sampling sites (Figure 2) and 65 ASVs (36%) were shared between mid-lake and littoral sites (in total 90.1% shared ASVs). Mid-lake and embayment sites shared three ASVs, and littoral and embayment sites shared 55 ASVs (Figure 2). The most common seven bacterial phyla were Proteobacteria, Bacteroidetes, Acidobacteria, Fibrobacteres, Verrucomicrobia and Planctomycetes. The relative abundance of cyanobacteria in surface sediments was low, accounting for 0.14–0.59% of the total bacterial community (Figure 3a). Nine unique cyanobacteria ASVs were detected, representing four genera; *Snowella*, *Planktothrix*, *Dolichospermum* and *Cyanobium* (Figure 3b). Contrary to expectations based on *Microcystis* sp. specific 16S rRNA gene analyses, no *Microcystis* ASVs were detected (Figure 3b). Further integration of the raw data showed that *Microcystis* ASVs were present in very low numbers, but because they represented <0.01% of all ASVs, they were discarded in the bioinformatic pipeline.

**Figure 4:**
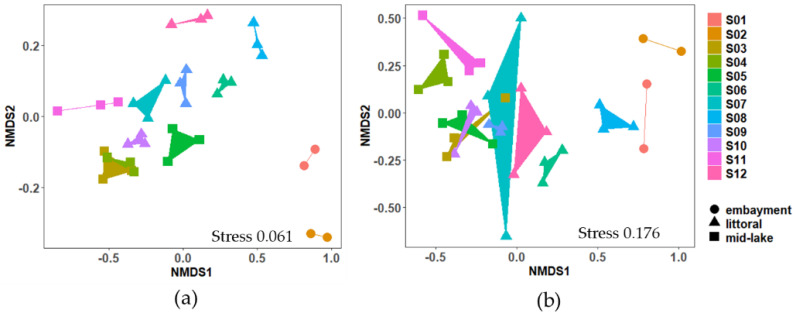
Two-dimensional non-metric multidimensional scaling (NMDS) ordination of microbial communities in the surface sediment samples (S01–S12). Plots are based on Bray–Curtis dissimilarities of 16S rRNA sequences of the; (**a**) total bacterial community, and (**b**) cyanobacterial community. Sampling site is indicated by shape (circle, triangle, square). Each point represents a sample. Replicates form a polygon. Stress value indicates fit of ordination, values ≤0.1 are considered fair, values ≤0.05 indicate good fit.

The NMDS ordination showed clear grouping of the total bacterial community by sampling site (Figure 4). The communities in the embayment sites (S01 and S02) were significantly different from littoral and mid-lake sites (S03–S12), (one-way distance-based permutational analysis of variance (PERMANOVA), emb.-littoral: *t* = 3.142, *p* = 0.0001; emb.-mid.: *t* = 4.011 *p* = 0.0007; mid.-littoral: *t* = 2.285, *p* = 0.0001; Figure 4a). Cyanobacterial communities were also significantly different between sites (PERMANOVA, emb.-littoral: *t* = 2.153, *p* = 0.001; emb.-mid.: *t* = 5.577, *p* = 0.0006; mid.-littoral: *t* = 3.026, *p* = 0.0001; Figure 4a).

#### 2.2.3. Toxin Analysis

MCs were not detected (<0.02 ng mL^−1^ or <2 µg g^−1^ wet weight in a 10 g sample) in any of the surface sediments collected. Recovery rates of the spiked MC congeners ranged between 25% for MC-RR, 73% for MC-LR and 126% for MC-LA (average result for the four spiked samples), indicating absorption to the sample matrix for certain congeners (Table S2). Recovery rates also differed between the four samples fortified with MCs, reinforcing this observation.

### 2.3. Sediment Cores

#### 2.3.1. Bacterial Community and Detection of *Microcystis* Sp. Specific 16S rRNA

**Figure 5:**
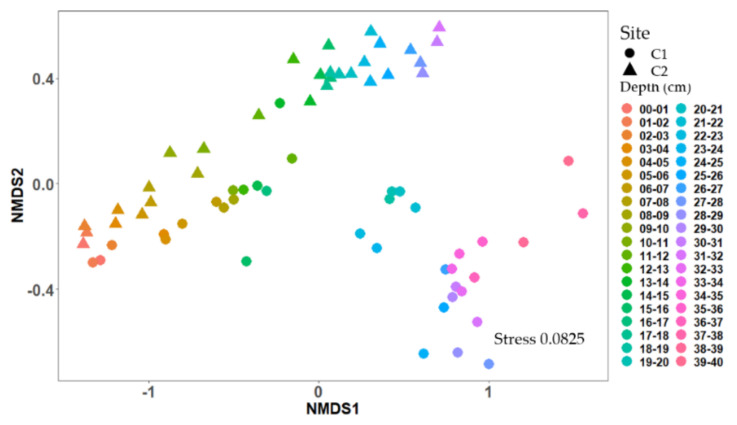
Two-dimensional non-metric multidimensional scaling (NMDS) ordination of bacterial community of sediment cores. Plots are based on Bray–Curtis similarities of 16S rRNA sequences of the total bacterial community. Color indicates depth layer, sampling site is indicated by shape (circles = C1 (mid-lake), triangles = C2 (littoral)). Each point represents a sample.

**Figure 6:**
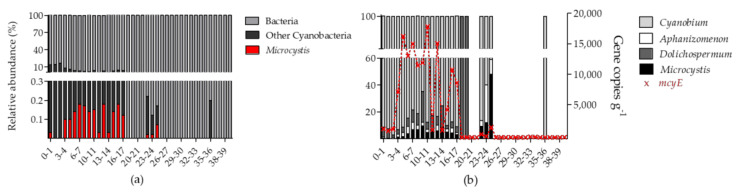
Bacterial community of the sediment core layers (cm) from the mid-lake (C1). Relative abundance of; (**a**) *Microcystis* and other cyanobacteria in the total bacterial community, and (**b**) Cyanobacteria genera in the total cyanobacteria community based on 16S rRNA sequences. *mcyE* gene copies (g^−1^ sediment) were detected with droplet digital PCR. Missing bars indicate that cyanobacterial taxa accounted for <0.1% of the total bacterial community in these samples.

High throughput sequencing of 16S rRNA amplicons of sediment core samples resulted in a total of 1712 unique ASVs of which 1521 (89%) were shared among both cores (Figure S1). An additional 159 ASVs occurred only in the mid-lake core, and 32 ASVs were unique to the littoral core (Figure S1). NMDS ordination of the total bacterial community shifted with increasing depth in both cores, indicating an increasing dissimilarity between communities with increasing depth (Figure 5). In total, nine ASVs were assigned to cyanobacteria representing four genera *Microcystis*, *Aphanizomenon*, *Dolichospermum* and *Cyanobium* (Figure 6b). In the mid-lake core, the cyanobacterial ASVs occurred mainly in the upper 17 cm (0.4–16.5% relative abundance) and in the littoral core, in the upper 12 cm (0.3–10.4% relative abundance), showing in both cores a decreasing trend in relative abundance with increasing sediment depth (Figure 6a and Figure S1a). In deeper sediment core layers, cyanobacterial ASV abundance was below 0.1% of total bacterial community (Figure 6a and Figure S1a).

In the littoral core *Microcystis* was detected only in traces in the uppermost layer (0–1 cm, 0.03% Figure 5a), but was not detected in the upper 1–3 cm. No *Microcystis* was detected in the upper 0–3 cm of littoral core (Figure S1). Cyanobacterial taxa found in these upper layers belonged to the genera of *Dolichospermum*, *Cyanobium* and *Aphanizomenon* (Figure 5b and Figure S2). In the mid-lake core, *Microcystis* was first detected at 4 cm depth and was present to a depth of 25 cm (Figure 5a). *Microcystis* abundance ranged between <1% to 48% of the cyanobacterial community accounting for 0–0.18% of the total bacterial community in the sediment layers (Figure 5). A peak in the relative abundance for *Microcystis* was observed at 22–25 cm depth, accounting for 9%, 12% and 48% of the cyanobacterial community, respectively. In the littoral core, *Microcystis* was first detected at 5 cm. The relative abundance of *Microcystis* in the cyanobacterial community was much lower compared to the mid-lake core accounting for ≤14% (Figure S2).

#### 2.3.2. Detection of *Microcystis* Specific *mcyE*

The vertical profile of *mcyE* copy numbers in the mid-lake core showed a similar pattern to the sequencing results (Figure 5b). Copy numbers of *mcyE* were detected in lower numbers in the upper 3 cm of the core (1089–1361 copies g^−1^ sediment) but increased from 4 to 17 cm. A peak concentration of 18,217 *mcyE* copies g^−1^ sediment was observed at 10–11 cm. Beyond 17 cm, *mcyE* copy numbers were not detected (<100 copies g^−1^ sediment).

#### 2.3.3. Sediment Chronology

**Figure 7:**
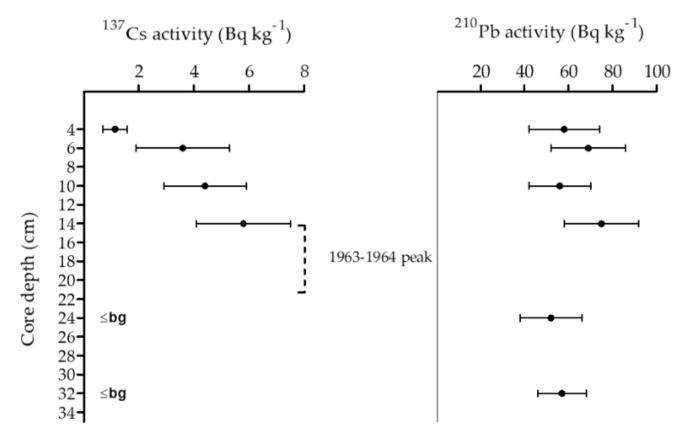
Radionuclide profile of ^137^Cs and ^210^Pb of the sediment core from the mid-lake (C1). Supported ^210^Pb (^226^Rd) has been subtracted from plotted ^210^Pb data. Measured value consistent with the background measurement are indicated with (≤bg). Error bars indicate uncertainty based on the combined standard uncertainty multiplied by a coverage factor providing a level of confidence of 95%. “1963-1964 peak” refers to the presumed marker position of the ^137^Cs maximum from 1963–1964.

The ^137^Cs chronomarker profile showed a typical increase in activity over sediment depth. The increase was from 4 cm sediment depth down to a depth of 14–15 cm and was below background level for the two deeper layers (24–25 cm and 32–33 cm; Figure 7). The 1963–1964 (nuclear weapon testing) ^137^Cs activity peak was not directly measured. However, the peak was assumed to be located between the highest ^137^Cs activity (14 cm depth) and the first below background measurement (24 cm; Figure 7). Due to the low temporal resolution, no dating model was applicable to this ^137^Cs profile. ^210^Pb activity showed a non-linear activity pattern with no clear increasing or decreasing trend. Therefore, the pattern could not be interpreted as a function of radioactive decay and no ^210^Pb dating model could be applied.

## 3. Discussion

### 3.1. Bacterial Community and Microcystis Abundance in Surface Lake Sediment

In this study, we initially set out to explore the spatial variability of *Microcystis* and other cyanobacteria in surface sediments to improve knowledge of fine-scale distribution patterns needed for paleolimnological research. Initially, we planned to explore only the cyanobacterial component of the metabarcoding data but given the unexpectedly low abundance of cyanobacteria, we expanded our analysis to the entire bacterial community. This also gives these results greater relevance to paleolimnological studies which are now incorporating molecular microbial analysis into their suite of techniques [23,24,35].

There are only a few studies available which focus on spatial variability of sediment DNA on a fine scale. O’Donnell et al., [36] assessed a near-shore marine habitat and observed a decrease in similarity of sediment microbial communities with increasing distance between sampling sites. To our knowledge there is just one other paleolimnological study focusing on historic cyanobacteria communities which sampled several cores in the same lake [37]. However, the latter study focused on comparing the quality and integrity of a preserved DNA fragments between cores, rather than characterizing the cyanobacterial community.

The results of the present study demonstrate that surface sediment samples taken from a central point in the lake capture approximately 90% of the microbial diversity in the mid-lake and littoral zones. This is especially important as most paleolimnological studies use single sediment cores from the deepest point in a lake. In contrast, the bacterial communities in the embayment sites were significantly different to the littoral and mid-lake sites and varied markedly among each other. This is most likely due to differences between abiotic parameters compared to littoral and mid-lake sites as reflected by lower dissolved oxygen and water depth. In addition, the different physical and chemical sediment characteristics might have an impact on DNA preservation between the embayment sites [19]. However, correlating differences in environmental parameters to differences of bacterial community structure was not the focus of this study and requires a more comprehensive sampling approach. It is important to recognize that Lake Rotorua is a small lake. In larger lakes, a higher spatial diversity of abiotic parameters and bacterial communities might be expected.

In the present study we expected to observe a high number of *Microcystis* 16S rRNA and *mcyE* gene copies and MCs in the surface sediment samples. This assumption was based on the occurrence of intense *Microcystis* blooms recorded within the last decade [21,38] and the occurrence of high numbers of *Microcystis* cells on surface sediments during a study undertaken in 2014 [21]. *Microcystis* can overwinter on the sediment surface [22,39,40]. The overwintering cells can resist harsh environmental conditions and form an inoculum for new spring blooms [41,42]. The abundance of overwintering cells has been shown to vary spatially, with higher abundance generally occurring at greater depths [21,41]. However, in the present study, only traces of *Microcystis* 16S rRNA gene copies and none of the eleven MCs analyzed were detected in the sediment surface layers. The absence of *Microcystis* and MCs in the surface sediment, despite the recent reports of MC-producing *Microcystis* in the lake, prompted us to take sediment cores to ensure that our detection methods were viable and that DNA and toxins were preserved in the sediment of Lake Rotorua.

### 3.2. Historic Microcystis Populations

In agreement with the results from surface sediments, *Microcystis* 16S rRNA and *mcyE* gene copies were absent in the upper 3 cm of the sediment cores. However, *Microcystis* 16S rRNA and *mcyE* gene copies were detected in greater depths indicating that toxin producing strains of this species have been present in the lake since around the 1950s. The reasons why there was no *Microcystis* and a generally low abundance of cyanobacteria before the 1950s cannot be readily explained with the data presented here. It might be linked to changes in land use of the lake catchment. The land around the lake was once podocarp forest, but this was burnt and replaced by pasture. Together with the rising usage of fertilizers, this has caused significant nutrient enrichments in the region’s waterways [43]. An increase of cyanobacteria during the 1900s was also observed in other lake sediment studies of North America [4] and Europe [6] and was linked to nutrient enrichment as a result of intensified agriculture.

Differences in historic bacterial communities between the littoral and mid-lake cores are probably due to water level fluctuations of Lake Rotorua. We assume that the current littoral area for the lake may have been swamp or wetland for some time or even for reoccurring time periods. This was supported by the fact that we were unable to obtain a longer core as we hit what we presume was thick historic vegetation and root. The mid-lake core is therefore more representative for looking at the historic bacterial communities of Lake Rotorua.

### 3.3. Why Is Microcystis Absent in the Surface Sediments?

The absence of *Microcystis* 16S rRNA and *mcyE* gene copies and MCs in the uppermost layers of all sediment samples of Lake Rotorua, corresponds with the observation that since 2016 (July and October 2017, January 2018) no *Microcystis* cells have been recorded in water column samples (Environment Canterbury, unpublished monitoring data). In November 2016, an earthquake of magnitude 7.8 struck the Kaikoura region. This was the second biggest earthquake recorded in New Zealand since European settlement. The earthquake triggered an estimated 100,000 landslides in the region, although no major ones were visible directly around the lake [44,45]. It is possible that this event resulted in a significant flux of sediment into the lake. Lake sediments have previously been used to reconstruct the chronology of natural disasters including floods [46] and earthquakes [47,48]. Earthquakes are linked to increased sediment fluxes to rivers and basins as a consequence of seismically induced landslides which often leads to a short-term increase of local and distant erosion rates [49]. These sediment fluxes can be identified as lake deposits (turbidites), which differ in structure from sediments originating from regular sedimentation processes, and can help to reconstruct long-term earthquake occurrences as previously shown for the New Zealand alpine fault [50].

While the ^137^Cs profile of the sediment core was as expected, ^210^Pb did not display its typical linear decay profile known from undisturbed sediments. Non-linear ^210^Pb profiles where previously shown for Lake Antern in the French Alps [51] and the Chilean lake district [52] as a result of earthquake-induced sediment changes. These changes can result from direct seismological mixing of old sediment layers containing ^210^Pb depleted material [51], or from landslides triggered by earthquakes, which increase erosion rates and sediment fluxes to rivers and basins [49], thereby overlaying lake sediments. The thickness of the earthquake-induced mass movement deposits varies between lakes and depends on the magnitude of the seismic event, site and size of triggered landslides as well as on the amount and quality of the erodible material in the catchment area. Of the few studies that have linked lake sediment deposits to the intensity of historic earthquake events, seismic event-linked deposits in lake sediment cores from the European Alps ranged between 1 to >20 cm thickness [47,51], in a Chilean lake between 0.5 to 7 cm [48] and in alpine lakes of New Zealand between 0.2 to 20 cm [50]. This demonstrates that each lake needs to be analyzed individually to allow any deduction of the impact of the seismic event on lake sediments.

The 2016 Kaikoura earthquake had a magnitude of 7.8, which was higher or equivalent to the seismic event related changes in the sediments described for the European Alps, Chile and earlier seismic events in New Zealand. Thus, it is likely that the disturbed upper 3–4 cm in Lake Rotorua resulted from a mixing effect as well an increased deposition of erosion material [51]. The finding that other cyanobacterial taxa were detected in the upper 3 cm and intact biofilms were found at the sediment surface (Figure S3), may appear contradictory to the disturbed sediment hypothesis. However, *Cyanobium* and *Snowella* are common freshwater picocyanobacteria [53]. They are known to be very adaptive to changing environmental conditions [54,55] and may represent the pioneering species occupying new niches after major environmental changes induced by increased sediment fluxes. *Aphanizomenon* and *Dolichospermum* both produce akinetes, thick walled cells that remain dormant until environmental conditions are favorable. It is possible that these were more resilient to sediment mixing than *Microcystis* vegetative cells or survived in shoreline terrestrial habitats, allowing these genera to rapidly recolonize following the sediment disturbance.

## 4. Conclusions

In this study, we demonstrate that bacterial communities of Lake Rotorua surface sediments significantly differed between lake sites (mid, littoral and embayment). However, the majority of ASV were shared between the mid and littoral sites, indicating that in small to medium sized lakes, the sampling approach used by most paleolimnological studies will also capture dominant microbial communities. The absence of *Microcystis* 16S rRNA and *mcyE* gene copies and MCs in surface sediments, in addition to the disturbed ^210^Pb profile, suggests that allochthonous processes may have impacted the surface sediment, changing the cyanobacterial communities. The 2016 Kaikoura earthquake may have played a role in this, but further research is required to confirm this hypothesis. The absence of *Microcystis* 16S rRNA and *mcyE* gene copies in the sediment layers prior to about 1950′s indicates that toxin producing cyanobacterial blooms in Lake Rotorua are a relatively recent occurrence.

## 5. Materials and Methods

### 5.1. Study Site

**Figure 8:**
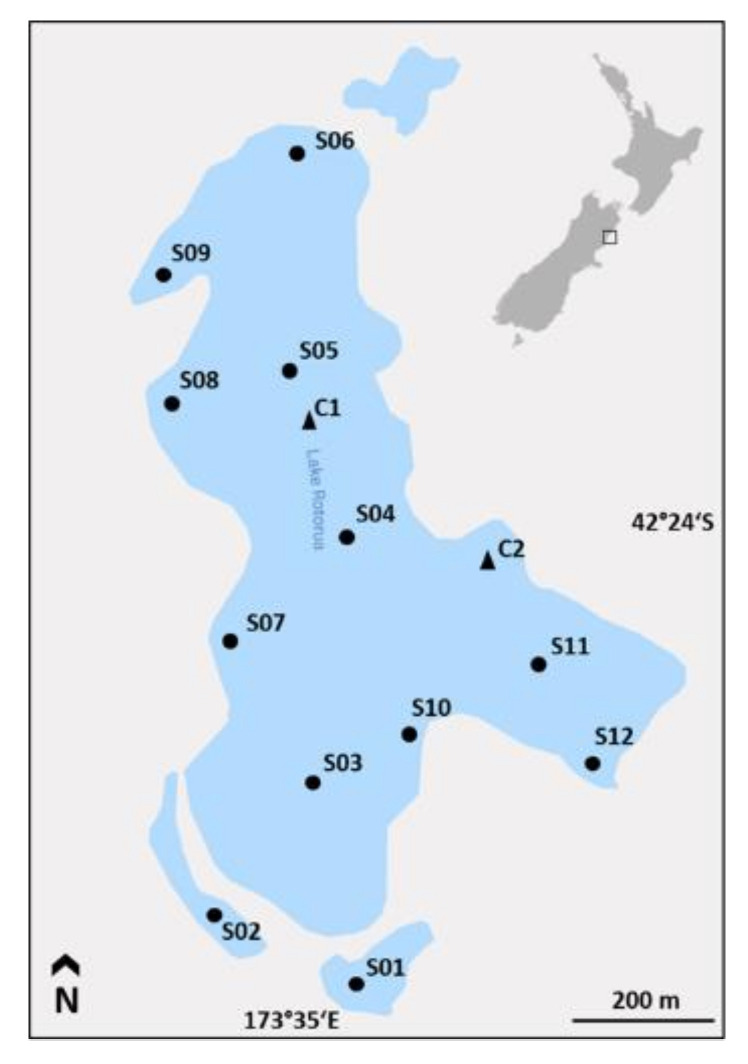
Map of Lake Rotorua (Kaikoura, New Zealand) and the 14 sediment sampling sites (12 surface sediments (S01–S12) and two sediment cores (C1 and C2)). Inset: map of New Zealand showing site of the lake.

Lake Rotorua (42°24′05″ S, 173°34′57” E) is a small (0.55 km^2^), shallow (max. depth 3 m), hypertrophic lake in the northeast of the South Island of New Zealand [34] (Figure 8). The average temperature in the nearby town of Kaikoura is 12.7 °C and precipitation averages 881 mm per year. In the early 1900s the native podocarp-mixed hardwood forest that dominated the catchment was removed. The catchment now has a mixed land use of low intensity grazing, native scrub and bushland, and exotic weeds and trees. Inflow comes largely from surface run-off during rainfall events. There is a single small outflow at the southern end of the lake, and the lake has an estimated residence of 0.86 y^−1^ [56]. During a two year monitoring study, Lake Rotorua was shown to experience annual cyanobacterial blooms between spring and autumn, with seasonal succession from nitrogen fixers (*Dolichospermum* and *Aphanizomenon*) to *Microcystis* later in summer [38]. During *Microcystis* blooms, peak concentrations of >2000 µg L^−1^ total MC and 4 × 106 cells mL^−1^ have been measured [38,57].

### 5.2. Sediment Sampling

Surface sediment samples were collected at 12 sites in January 2018 using a sediment core sampler (UWITEC, Mondsee, Austria; Figure 8, Table 1). Surface samples were collected in embayments (S01 and S02), mid-lake (S03-05, S11) and littoral zones (S06-10, S12) from the top layer (0.5 cm) of the core. At each site, three separate surface samples were taken. At two sites, sediment cores were retrieved; (i) C1, collected mid-lake (40 cm length), and (ii) C2, collected in the littoral zone (32 cm length). Sediment cores were sectioned into 1 cm increments. Sediment subsamples for DNA extraction were stored at −20 °C until further processing. Subsamples for toxin extraction and age dating were sampled into 50 mL Falcon tubes and stored at −20 °C until analysis. At each sampling site the water temperature, pH, salinity and dissolved oxygen were measured using a YSI water quality sonde (YSI, Yellow Springs, OH, USA).

### 5.3. DNA Extraction and Inhibition Test

Subsamples of sediment (0.2–0.4 g) were weighed into the first tube of a PowerSoil DNA Isolation Kit (Qiagen, Germantown, MD, USA) and DNA extracted according to the protocol supplied by the manufacturer. A random selection of DNA samples (*n* = 10) were screened in duplicates for inhibition using an internal control quantitative PCR (qPCR) assay [58].

### 5.4. Quantitative PCR for Microcystis and mcyE Enumeration in Surface Sediments

The number of toxin producing *Microcystis* cells present was determined by amplifying the *mcyE* gene (a single copy gene) and quantifying the number of amplicons, using a standard curve generated from MC-producing *Microcystis* sp. strain CAWBG617 [38]. The qPCR was performed in triplicate for each sample in a 12.5 μL of reaction mix containing 6.25 µL KAPA Probe Fast qPCR Kit Master Mix (2×), 1 µL of primers targeting a region within the *mcyE* open reading frame (0.4 µM, McyE-F2 and MicMcyE-R8) [59], 0.2 µL of *mcyE* probe [60] and 1 µL of template DNA per sample. The standard curve was constructed using a purified (AxyPrep PCR Clean-up Kit, Axygen Biosciences, Union City, CA, USA) PCR product generated with the primers described above. The number of copies in the PCR product used for the standard curves was determined using: (A × 6.022 × 1023)/(B × 1×109 × 650), with A being the concentration of the PCR product, 6.022 × 1023 (Avogadro’s number), B being the length of the PCR product, 1 × 109 used to convert to ng, and 650 the average molecular weight per base pair (bp). Each point of the standard curve was analyzed in triplicate for each qPCR run conducted. The standard curve generated was linear (*R*^2^ > 0.99) and PCR efficiency was >0.9. The results of copies per μL DNA were converted to copies per gram of sediment using: (D × 80)/E, with D being the number of copies per μL, × 80 since 1 μL DNA of the elution volume (80 μL) was used, and E being the weight of sediment (g) used for DNA extraction.

To detect and quantify *Microcystis* sp., a region of the 16S rRNA gene was amplified using *Microcystis* sp. specific primers MICR184F [61] and MICR431R [62]. qPCR and standard curve construction was performed as described for *mcyE* enumeration.

### 5.5. Droplet Digital PCR for mcyE Enumeration in Sediment Cores

Quantification of the *mcyE* copy numbers in sediment cores was carried out with droplet digital PCR (ddPCR) using a BioRad QX200 system. Each ddPCR reaction included 2 μL of 900 nM of each primer and 0.55 μL of 250 nM of primer probe (primers and probe as described for qPCR), 11 μL of BioRad ddPCR Supermix for probes, 1.5 μL DNA, and 4.95 μL of sterile water. The reaction mixture was divided into nanodroplets by joining 20 μL of the reaction mixture with 70 μL of BioRad droplet oil. After processing, the nanodroplet volume resulted in a total of 40 μL, which was transferred to a PCR plate for amplification at 96 °C for 10 min, followed by 40 cycles at 94 °C for 30 s, 60 °C for 60 s and a final extension step of 96 °C for 10 min. The ddPCR was run on one plate, with four negative controls (containing all reagents plus DNA/RNA-free water) and four positive controls (genomic DNA extracted from a sample known to contain the *mcyE* gene) included. The results were converted to copies per gram of sediment using: (D × 66)/E, with D being the number of copies per μL, × 66 since 1.5 μL DNA of the elution volume (100 μL) was used, and E being the weight of sediment (g) used for DNA extraction.

### 5.6. High Throughput Sequencing

#### 5.6.1. 16S rRNA PCR

Surface and core sediments were analysed via Illumina™ sequencing. A region of the 16S rRNA gene approx. 400 bp long was amplified using bacteria-specific primers 341F: 5′-CCT ACG GGN GGC WGC AG-3′ and 805R: 5′-GAC TAC HVG GGT ATC TAA TCC-3′, modified to include Illumina™ adapters [63,64]. PCR reactions were performed in 50 µL volumes containing 25 μL of AmpliTaq Gold^®^ 360 master mix (Life Technologies, Pleasanton, CA, USA), 12.5 μL GC inhibitor (Life Technologies, Pleasanton, CA, USA), 2 μL of each primer (10 μM, Integrated DNA Technologies, Coralville, IA, USA), 2 μL template DNA (between 60 and 220 ng) and 6.5 μL Milli-Q water. PCR cycling conditions were: 95 °C for 10 min, followed by 27 cycles of 95 °C for 30 s, 50 °C for 30 s, 72 °C for 45 s, and a final extension of 72 °C for 7 min. PCR products were visualized with 1.5% agarose gel electrophoresis with Red Safe DNA Loading Dye (iNtRON Biotechnology Inc, Kyungki-Do, Korea) and UV illumination to ensure amplification of a single 400 bp product. PCR products were purified (Agencourt^®^ AMPure^®^ XP Kit; Beckman Coulter, CA, USA), quantified (Qubit^®^ 20 Fluorometer, Invitrogen), diluted to 10 ng µL^−1^ and submitted to New Zealand Genomics Limited (Auckland, New Zealand) for library preparation. Sequencing adapters and sample-specific indices were added to each amplicon via a second round of PCR using the NexteraTM Index kit (IlluminaTM, Melbourne, Australia). Amplicons were pooled into a single library and paired-end sequences (2 × 250) were generated on a MiSeq instrument using the TruSeqTM SBS kit (IlluminaTM, Melbourne, Australia). Sequence data were automatically demultiplexed using MiSeq Reporter (v2), and forward and reverse reads were assigned to samples.

#### 5.6.2. Quality Control and Taxonomic Assignment of Illumina Sequences

Analysis of sequencing data was performed using the QIIME 2 2019.1 [65]. Raw sequence reads were demultiplexed and quality filtered using q2-demux plugin. Denoising of sequences was followed by chimera identification and removal using DADA2 [66]. To account for differential sequencing depth among samples, the number of reads per sample was rarefied to 28,700 (surface sediment samples) and 26,177 reads (sediment core samples). Five samples were excluded from further analysis because of low read number; two surface sediments (S01 and S02), two subsamples from the mid-lake sediment core (9–10 cm and 17–18 cm) and one subsample from the littoral core (29–30 cm). The implemented q2-feature-classifier-plugin (a scikit-learn naive Bayes machine-learning classifier) [67] was used for classification and pre-trained on the SILVA bacteria 16S rRNA gene reference database (version 132) [68], trimmed with the primer sequences prior to taxonomic assignment. Quality filtered and taxa assigned ASVs were further pre-processed and filtered using R (version 3.6.1, R Foundation for Statistical Computing, Vienna, Austria) [69] and the phyloseq package [70]. Chloroplast, rare ASVs accounting for less than 0.1% of the total bacterial community, and ASVs which could not be assigned further than kingdom level were excluded from further analysis.

#### 5.6.3. Graphics and Data Analysis of High Throughput-Sequences

To visualize bacterial community dissimilarities between sampling sites and depth increments, non-metric-multidimensional-scaling (NMDS) was performed, using the R vegan package [71]. Filtered and pre-processed ASVs were previously fourth root transformed and a Bray–Curtis dissimilarity matrix was calculated. To test for significance of observed dissimilarities in bacterial community between sites, a PERMANOVA was performed using PRIMER (version 7, PRIMER-e, Auckland, New Zealand) with the PERMANOVA + add on [72]. Venn diagrams were designed using the Venny tool version 2.1 [73]. GraphPad prism (version 5, San Diego, CA, USA) for Windows was used for all other figure preparations.

### 5.7. Toxin Analysis

#### 5.7.1. Toxin Extraction from Surface Sediments

Surface sediment samples (6–10 g wet weight) were freeze-dried and extracted with three consecutive extractions using 100% MeOH (5 mL each). During each extraction step, samples were vortexed for 1 min, sonicated for 15 min and centrifuged (3200× *g*, 10 min, 10 °C). The extracts were pooled and dried at 40 °C under a stream of nitrogen gas and dissolved in 1 mL 100% MeOH. Extracts were stored at −20 °C until measurement.

#### 5.7.2. Toxin Analysis of Surface Sediments

Toxin extracts were analyzed by ultra-performance liquid chromatography-tandem mass spectrometry (UPLC-MS/MS) as described previously [74]. To assess the extraction method and check for matrix effects, the recovery rate of MC was determined by fortification with a semi-purified extract of *Microcystis* CAWBG11 which produces a range of MC congeners [75]. The MC-enriched extract (dissolved in 80% MeOH) was added to sediment samples and incubated at 4 °C for 30 min. Samples were extracted as described previously and analyzed via UPLC-MS/MS.

### 5.8. Sediment Chronology of Mid-Lake Sediment Deep Core

For age-dating of the mid-lake sediment core, subsamples representing six increments (4–5, 6–7, 10–11, 14–15, 24–25 and 32–33 cm) were analyzed using gamma spectrometry of the long-lived radionuclides ^210^Pb and ^137^Cs at the Institute of Environmental Science and Research (Christchurch, New Zealand). Activities were quantified using a gamma counter with a high-purity germanium well detector. Activities are reported in Bq per kg. Uncertainties are based on the combined standard uncertainty multiplied by a coverage factor (k) = 2, (providing a level of confidence of 95%) as described before [76,77]. Core chronologies from ^210^Pb and ^137^Cs measurements were calculated using the constant rate of supply (CRS) model in R by means of the mudata2 package [78]. Assuming that the supply of unsupported ^210^Pb to the sediment is the same for each time interval, the CRS model is calculated as follows:

tz = λ^−1^ − 1 ln(A_0_ Az^−1^) (1)

where (t) is the age of any interval (z) [79].

## Figures and Tables

**Table 1:** Overview of sediment samples taken in this study.

	Surface Sediments												Sediment Cores	
Sample name	S01	S02	S03	S04	S05	S06	S07	S08	S09	S10	S11	S12	C1	C2
Sediment depth (cm)	0–0.5												0-40	0-32
Replicates	3												1	
Increments	1												40	32
Site location	embayment		mid-lake			littoral					mid-lake	littoral	mid-lake	littoral

## Supplementary Materials

The following are available online at https://www.mdpi.com/2072-6651/12/9/580/s1. Table S1: abiotic parameters of surface sediment sampling sites, Table S2: recovery rates of microcystin (MC) congeners, Figure S1: Venn diagram illustrating the unique and shared amplicon sequence variant (ASV) between the two sediment cores from the mid-lake (C1) and the littoral (C2), Figure S2: bacterial community of the sediment core from the mid-lake (C2). Relative abundance of; (a) *Microcystis* and other cyanobacteria in the total bacterial community, and (b) Cyanobacteria genera in the total cyanobacteria community based on 16S rRNA sequences. Missing bars indicate that cyanobacterial taxa accounted for <0.1% of the total bacterial community in these samples, Figure S3: biofilm growing on the surface sediment of Lake Rotorua.
